# Hirshfeld analysis and mol­ecular docking with the RDR enzyme of 2-(5-chloro-2-oxoindolin-3-yl­idene)-*N*-methyl­hydrazinecarbo­thio­amide

**DOI:** 10.1107/S2056989017005461

**Published:** 2017-04-13

**Authors:** Jecika Maciel Velasques, Vanessa Carratu Gervini, Lisliane Kickofel, Renan Lira de Farias, Adriano Bof de Oliveira

**Affiliations:** a Universidade Federal do Rio Grande (FURG), Escola de Química e Alimentos, Rio Grande, Brazil; b Universidade Estadual Paulista (UNESP), Instituto de Química, Araraquara, Brazil; c Universidade Federal de Sergipe (UFS), Departamento de Química, São Cristóvão, Brazil

**Keywords:** crystal structure, isatin thio­semicarbazone derivative, hydrogen bonding, Hirshfeld surface analysis, RDR-thio­semicarbazone *in silico* evaluation

## Abstract

The acetic acid-catalyzed reaction between 5-chloro­isatin and 4-methyl­thio­semicarbazide yields the title compound, C_10_H_9_ClN_4_OS (I) (common name: 5-chloro­isatin-4-methyl­thio­semicarbazone). The mol­ecule is nearly planar (r.m.s. deviation = 0.047 Å for all non-H atoms), with a maximum deviation of 0.089 (1) Å for the O atom. An *S*(6) ring motif formed by an intra­molecular N—H⋯O hydrogen bond is observed. In the crystal, mol­ecules are linked by N—H⋯O hydrogen bonds, forming chains propagating along the *a*-axis direction. The chains are linked by N—H⋯S hydrogen bonds, forming a three-dimensional supra­molecular structure. The three-dimensional framework is reinforced by C—H⋯π inter­actions. The absolute structure of the mol­ecule in the crystal was determined by resonant scattering [Flack parameter = 0.006 (9)]. The crystal structure of the same compound, measured at 100 K, has been reported on previously [Qasem Ali *et al.* (2012 ▸). *Acta Cryst.* E**68**, o964–o965]. The Hirshfeld surface analysis indicates that the most important contributions for the crystal packing are the H⋯H (23.1%), H⋯C (18.4%), H⋯Cl (13.7%), H⋯S (12.0%) and H⋯O (11.3%) inter­actions. A mol­ecular docking evaluation of the title compound with the ribonucleoside diphosphate reductase (RDR) enzyme was carried out. The title compound (I) and the active site of the selected enzyme show Cl⋯H—C*(LYS140)*, *Cg*(aromatic ring)⋯H—C*(SER71)*, H⋯O—C*(GLU200)* and Fe^III^⋯O⋯Fe^III^ inter­molecular inter­actions, which suggests a solid theoretical structure–activity relationship.

## Abstract 2

The title isatin thio­semicarbazone derivative is an inter­mediate in the synthetic pathway of HIV-1 reverse transcriptase inhibitors. A mol­ecular docking evaluation of the title compound with the ribonucleoside diphosphate reductase (RDR) enzyme was carried out.

## Chemical context   

Methods for the synthesis of isatin derivatives were first reported in the first half of the 19th century (Erdmann, 1841*a* ▸,*b* ▸; Laurent, 1841 ▸), while for thio­semicarbazone derivatives one of the first reports can be traced back to the early 1900’s (Freund & Schander, 1902 ▸). Initially, thio­semi­carbazone chemistry was not related to the pharmacological sciences. This has changed since the discovery that *in vitro* assays of sulfur-containing compounds showed that they are effective for *Mycobacterium tuberculosis* growth inhibition (Domagk *et al.*, 1946 ▸). In the 1950’s, the synthesis of isatin–thio­semicarbazone derivatives was reported (Campaigne & Archer, 1952 ▸) and *in vitro* assays indicated such compounds to be active against Cruzain, Falcipain-2 and Rhodesian (Chiyanzu *et al.*, 2003 ▸). Nowadays, many isatin–thio­semi­carbazone derivatives employed in medicinal chemistry. For example, 1-[(2-methyl­benzimidazol-1-yl) meth­yl]-2-oxo-indo­lin-3-yl­idene]amino]­thio­urea is an *in vitro* and *in silico* Chikungunya virus inhibitor (Mishra *et al.*, 2016 ▸). The title compound (I), 5-chloro­isatin-4-methyl­thio­semicarbazone, is an inter­mediate in the synthetic pathway of HIV-1 (human immunodeficiency virus type 1) RT (reverse transcriptase) inhibitor synthesis (Meleddu *et al.*, 2017 ▸); a new crystal structure determination is reported here, the original work having been published by Qasem Ali *et al.* (2012 ▸). Thus, the crystal structure determination of isatin–thio­semicarbazone-based mol­ecules is an intensive research area in medicinal chemistry and the main focus of our work.
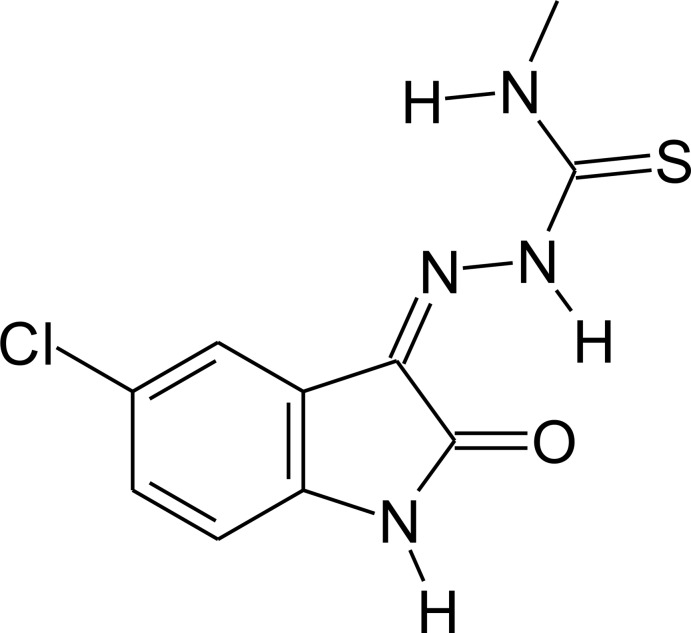


## Structural commentary   

**Figure 1:**
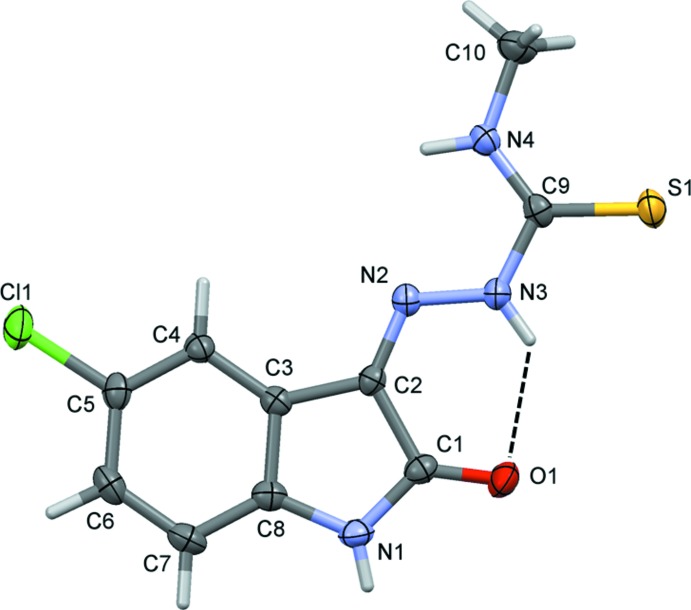
The mol­ecular structure of the title compound (I) (this work), showing the atom labelling and displacement ellipsoids drawn at the 50% probability level. The intra­molecular hydrogen bond [graph-set motif *S*(6)] is shown as a dashed line (see Table 1 ▸).

The present analysis of the title compound (I), measured at 200 K, is very similar to that measured by Qasem Ali *et al.* (2012 ▸) at 100 K. There is one intra­molecular hydrogen bond, N3—H3*N*⋯O1 (Table 1 ▸), with an *S*(6) graph-set motif (Fig. 1 ▸). The mol­ecule is almost planar (r.m.s. deviation = 0.047 Å for all non-H atoms), with maximum deviations of −0.089 (1), −0.073 (1) and 0.057 (1) Å for atoms O1, Cl1 and S1, respectively. In addition, the torsion angle for the N4—C9—N3—N2 unit is −0.8 (2)°.

## Supra­molecular features   

**Figure 2:**
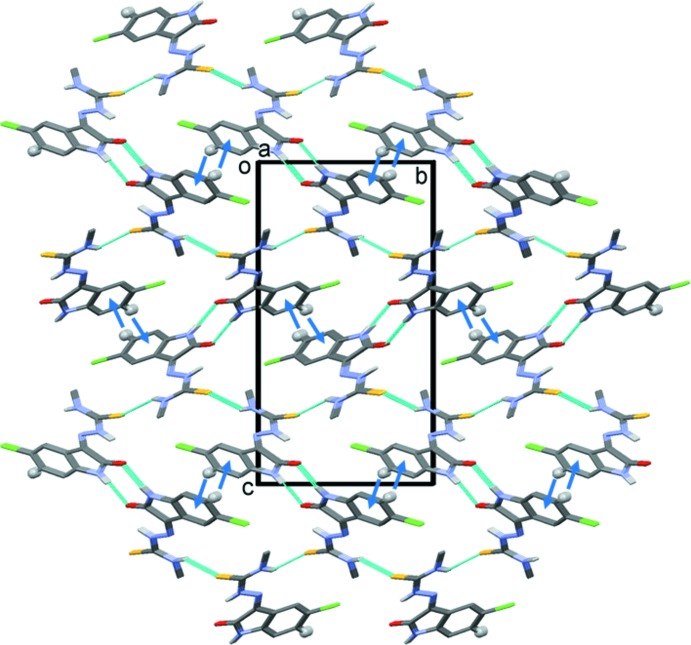
A view along the *a* axis of the crystal packing of the title compound (I) (this work). Details of the N—H⋯O and N—H⋯S hydrogen bonds (dashed lines) and the C—H⋯π inter­actions (blue arrows) are given in Table 1 ▸. H atoms not involved in these inter­actions have been omitted for clarity.

In the crystal, mol­ecules are linked by N1—H1*N*⋯O1^i^ hydrogen bonds, forming chains propagating along the *a*-axis direction. The chains are linked by N4—H4*N*⋯S^ii^ hydrogen bonds, forming a three-dimensional supra­molecular structure (Fig. 2 ▸, Table 1 ▸). The three-dimensional framework is reinforced by C6—H6⋯π^iii^ inter­actions, as shown in Fig. 2 ▸ (see also Table 1 ▸). The crystal structure determined in this work and that of the originally published article (Qasem Ali *et al.*, 2012 ▸) are, of course, similar.

## Hirshfeld surface analysis   

**Figure 3:**
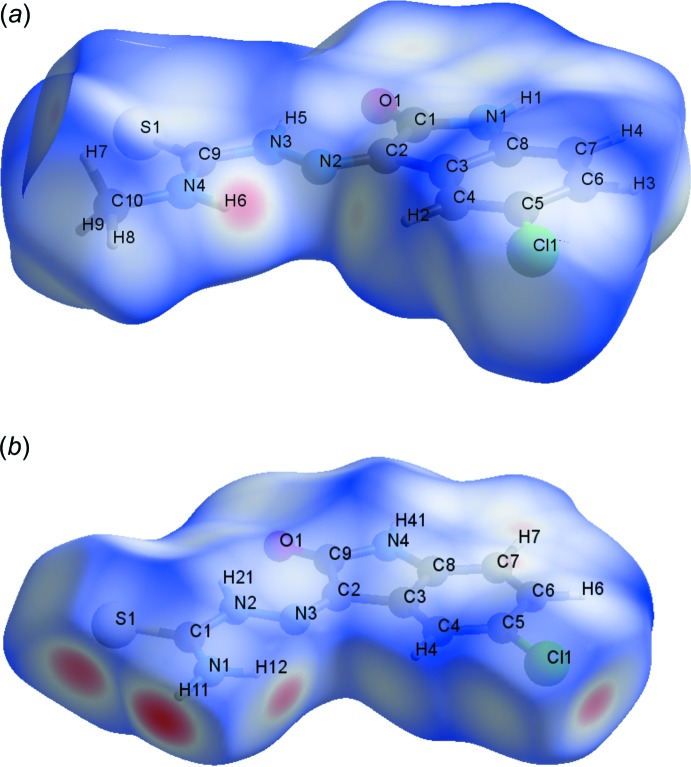
The Hirshfeld surface graphical representation (*d*_norm_) for the asymmetric unit of: (*a*) the title compound (I) (this work) and (*b*) 5-chloro­isatin-thio­semicarbazone (II) (de Bittencourt *et al.*, 2014 ▸). The surface regions with strongest inter­molecular inter­actions are drawn in magenta colour.

**Figure 4:**
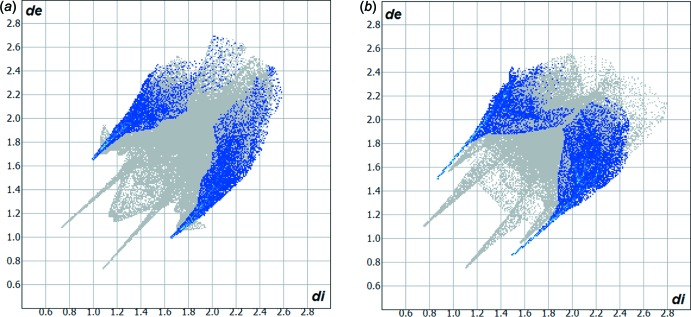
Hirshfeld surface two-dimensional fingerprint plots for the crystal structures of: (*a*) the title compound (I) (this work) and (*b*) 5-chloro­isatin-thio­semicarbazone (II) (de Bittencourt *et al.*, 2014 ▸), showing the H⋯S contacts in detail (cyan dots). The contribution of the H⋯S inter­actions to the mol­ecular cohesion of the crystal structures amounts to 12.0 and 17.2%, respectively. The *d*_e_ (*y* axis) and *d*_i_ (*x* axis) values are the closest external and inter­nal distances (Å) from given points on the Hirshfeld surface contacts.

The Hirshfeld surface analysis (Hirshfeld, 1977 ▸) of the crystal structure of (I) suggests that the contribution of the H⋯H inter­molecular inter­actions for the crystal structure cohesion amounts to 23.1%. The contributions of the other major inter­molecular inter­actions are: H⋯C (18.4%), H⋯Cl (13.7%), H⋯S (12.0%) and H⋯O (11.3%). The minor observed contributions for the crystal packing are H⋯N (5.3%) and C⋯N (4.2%). The Hirshfeld surface graphical representation, *d*_norm_, with transparency and labelled atoms indicates, in magenta, the locations of the strongest inter­molecular contacts, *e.g.* the H6 and H2 atoms, which are important for the inter­molecular hydrogen bonding (Fig. 3 ▸ *a*). The H⋯H, H⋯C, H⋯Cl, H⋯S and H⋯O contributions to the crystal packing are shown as a Hirshfeld surface two-dimensional fingerprint plot with cyan dots. The *d*_e_ (*y* axis) and *d*_i_ (*x* axis) values are the closest external and inter­nal distances (Å) from given points on the Hirshfeld surface contacts (Figs. 4 ▸ *a* and 5 ▸) (Wolff *et al.*, 2012 ▸).

## Mol­ecular docking   

Finally, for an inter­action between the 5-chloro­isatin-4-methyl­thio­semicarbazone (this work) and a biological target, the ribonucleoside diphosphate reductase (RDR), a lock-and-key supra­molecular analysis was carried out (Chen, 2015 ▸). The RDR enzyme was selected for this work due its importance in cell proliferation. It catalyzes the conversion of ribonucleotides to de­oxy­ribonucleotides, which is the rate-limiting step for DNA synthesis. In addition, a thio­semicarbazone derivative, the 3-amino-pyridine-2-carboxaldehyde thio­semi­carba­zone, shows RDR inhibition and biological activity is suggested by its coordination with the Fe ions of the enzyme active site (Popović-Bijelić *et al.*, 2011 ▸). The commercial name for this thio­semicarbazone derivative is Triapine. Its source until 2009 was Vion Pharmaceuticals Inc., New Haven, CT, United States. Since 2017, Trethera Corporation, Santa Monica, CA, and Nanotherapeutics Inc., Alachua, FL, have had a worldwide agreement for the development, production and commercialization of Triapine formulations and for its applications in hematological malignancies (Nanothera­peutics, 2017 ▸). This illustrates that academic institutions, public and private research facilities and industry have a high level of inter­est in thio­semicarbazone derivatives and in studies concerning RDR–thio­semicarbazone inter­actions.

The semi-empirical equilibrium energy of the title compound (this work) was obtained using the PM6 Hamil­tonian (Stewart, 2013 ▸), but the experimental bond lengths were conserved. The crystal structure of the RDR enzyme (PDB code: 1W68) was downloaded from the Protein Data Bank (Strand *et al.*, 2004 ▸). The calculated parameters were: heat of formation = 98.697 kcal mol^−1^, gradient normal = 0.68005, HOMO = −8.934 eV, LUMO = −1.598 eV and energy gap = 7.336 eV. The title compound (I) and the active site of the selected enzyme matches and structure–activity relationship can be assumed by the following observed inter­molecular inter­actions: Cl1⋯H—C*(LYS140)* = 2.538 Å, *Cg*(aromatic ring)⋯H—C*(SER71) =* 2.714 Å, H5⋯O—C*(GLU200) =* 1.663 Å, Fe1⋯O1 = 2.567 Å and Fe2⋯O1 = 2.511 Å. The *in silico* evaluation suggests through the graphical representation the bridging O1 atom connecting the two Fe^III^ metal centers by inter­molecular inter­actions (Fig. 6 ▸).

## Comparison with a related structure   

**Figure 5:**
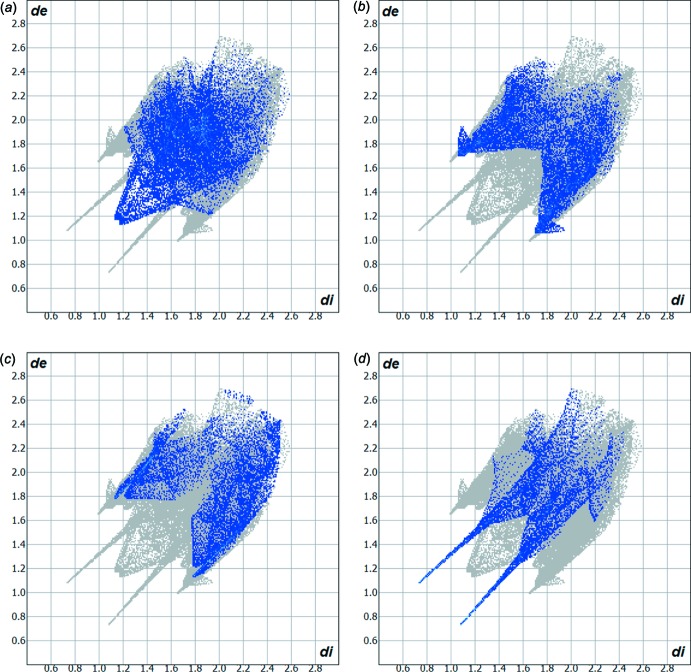
Hirshfeld surface two-dimensional fingerprint plots for the title compound (I) (this work), showing the (*a*) H⋯H, (*b*) H⋯C, (*c*) H⋯Cl and (*d*) H⋯O contacts in detail (cyan dots). The contributions of the inter­actions to the crystal packing amount to 23.1, 18.4, 13.7 and 11.3%, respectively. The *d*_e_ (*y* axis) and *d*_i_ (*x* axis) values are the closest external and inter­nal distances (Å) from given points on the Hirshfeld surface contacts.

**Figure 6:**
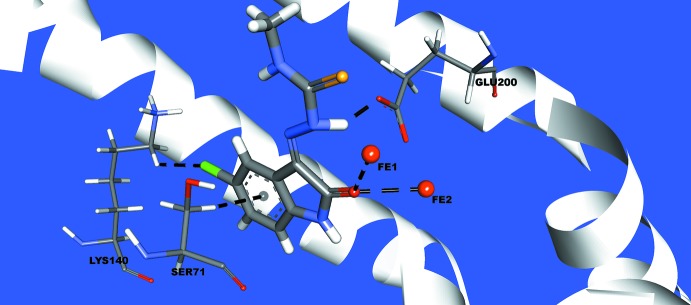
Graphical representation of a lock-and-key model for the title compound (I) (this work) and the RDR enzyme active site, with selected amino acid residues. The inter­actions are shown as dashed lines and the figure in the stick model is simplified for clarity.

**Figure 7:**
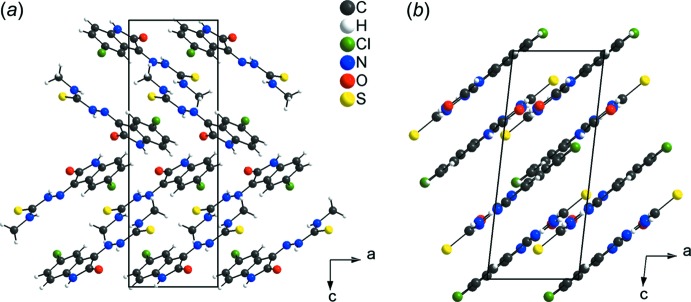
Section of the crystal structures of: (*a*) the title compound (I) (this work), and (*b*) 5-chloro­isatin–thio­semicarbazone (II) (de Bittencourt *et al.*, 2014 ▸), showing the mol­ecular stacking along the [001] direction. The crystal packing of both compounds is viewed along the *b* axis, and the figures are simplified for clarity.

Isatin–thio­semicarbazone derivatives have mol­ecular structural features in common, *viz.* nearly a planar geometry as a result of the *sp* ^2^-hybridized C and N atoms of the main fragment. For a comparison with the title compound [5-chloro­isatin-4-methyl­thio­semicarbazone (I); this work], 5-chloro­isatin-thio­semicarbazone, (II), was selected (de Bittencourt *et al.*, 2014 ▸) as both structures have the same main entity. The mol­ecular structural difference is the substitution of one H atom of the amine group of (II) by a methyl group in the title compound (I). Although the mol­ecular basis for the two compounds is the same, there are significant differences in the crystal packing. For compound (I), the unit cell is chiral and the mol­ecules are linked by hydrogen bonding into a three-dimensional network (Figs. 2 ▸ and 7 ▸ *a*), while for compound (II) the unit cell is centrosymmetric and the hydrogen bonding is observed in a planar arrangement, with the mol­ecules stacked along the [001] direction (Fig. 7 ▸ *b*). The terminal methyl group in (I) decreases the possibility of H-atom contacts with S and O acceptors, while in compound (II), the presence of the terminal unsubstituted amine increases the chances for hydrogen bonding, as suggested by the Hirshfeld surface analysis, *d*_norm_, for the two mol­ecules (Fig. 3 ▸ *a*,*b*). The Hirshfeld surface two-dimensional fingerprint plot shows that the contribution of the H⋯S inter­molecular inter­action to the crystal cohesion amounts to 12.0% in the title compound (I), while for the 5-chloro­isatin-thio­semicarbazone (II) it amounts to 17.2% (Fig. 5 ▸ *a*,*b*). The relationship between thio­semicarbazone derivatives, the mol­ecular assembly, the geometry of the H⋯S inter­actions and their contribution to the crystal structures can be seen in a recently published article (de Oliveira *et al.*, 2017 ▸).

## Synthesis and crystallization   

The starting materials are commercially available and were used without further purification. The synthesis of the title compound was adapted from a previously reported procedure (Freund & Schander, 1902 ▸). In an acetic acid-catalyzed reaction, a mixture of 5-cloroisatin (3 mmol) and 4-methyl-3-thio­semicarbazide (3 mmol) in ethanol (40 ml) was stirred and refluxed for 5 h. On cooling, a solid was obtained which was filtered off. Yellow prismatic crystals of the title compound were grown in tetra­hydro­furan by slow evaporation of the solvent.

## Refinement   

Crystal data, data collection and structure refinement details for the title compound (I) are summarized in Table 2 ▸. The NH H atoms were located in difference-Fourier maps and freely refined. The C-bound H atoms were positioned with idealized geometry and refined using a riding model: C—H = 0.95–0.98 Å with *U*_iso_(H) = 1.5*U*_eq_(C-meth­yl) and 1.2*U*_eq_(C) for other H atoms. The absolute structure of the mol­ecule in the crystal was determined by resonant scattering [Flack parameter = 0.006 (9)].

## Supplementary Material

Crystal structure: contains datablock(s) I, Global. DOI: 10.1107/S2056989017005461/su5363sup1.cif

Structure factors: contains datablock(s) I. DOI: 10.1107/S2056989017005461/su5363Isup2.hkl

Supporting information file. DOI: 10.1107/S2056989017005461/su5363Isup3.cml

CCDC reference: 1543340

Additional supporting information:  crystallographic information; 3D view; checkCIF report

## Figures and Tables

**Table 1:** Hydrogen-bond geometry (Å, °) *Cg* is the centroid of the C3–C8 ring.

*D*—H⋯*A*	*D*—H	H⋯*A*	*D*⋯*A*	*D*—H⋯*A*
N3—H3*N*⋯O1	0.83 (2)	2.12 (3)	2.756 (2)	134 (2)
N1—H1*N*⋯O1^i^	0.79 (2)	2.04 (3)	2.824 (2)	175 (2)
N4—H4*N*⋯S1^ii^	0.88 (3)	2.72 (3)	3.518 (2)	152 (2)
C6—H6⋯*Cg* ^iii^	0.95	2.61	3.410 (2)	142

Symmetry codes: (i) 
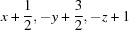
; (ii) 
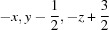
; (iii) 
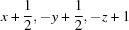
.

**Table 2:** Experimental details

Crystal data	
Chemical formula	C_10_H_9_ClN_4_OS
*M*_r_	268.72
Crystal system, space group	Orthorhombic, *P*2_1_2_1_2_1_
Temperature (K)	200
*a*, *b*, *c* (Å)	6.2584 (1), 10.1734 (2), 18.7183 (3)
*V* (Å^3^)	1191.78 (4)
*Z*	4
Radiation type	Mo *K*α
μ (mm^−1^)	0.48
Crystal size (mm)	0.46 × 0.16 × 0.12

Data collection	
Diffractometer	Bruker APEXII CCD area detector
Absorption correction	Multi-scan (*SADABS*; Bruker, 2014 ▸)
*T*_min_, *T*_max_	0.697, 0.749
No. of measured, independent and observed [*I* > 2σ(*I*)] reflections	10426, 2342, 2295
*R*_int_	0.013
(sin θ/λ)_max_ (Å^−1^)	0.617

Refinement	
*R*[*F* ^2^ > 2σ(*F* ^2^)], *wR*(*F* ^2^), *S*	0.020, 0.056, 1.05
No. of reflections	2342
No. of parameters	167
H-atom treatment	H atoms treated by a mixture of independent and constrained refinement
Δρ_max_, Δρ_min_ (e Å^−3^)	0.19, −0.15
Absolute structure	Flack *x* determined using 940 quotients [(*I* ^+^)−(*I* ^−^)]/[(*I* ^+^)+(*I* ^−^)] (Parsons *et al.*, 2013 ▸)
Absolute structure parameter	0.006 (9)

Computer programs: *APEX2* and *SAINT* (Bruker, 2014 ▸), *SHELXT2014* (Sheldrick, 2015*a* ▸), *SHELXL2016* (Sheldrick, 2015*b* ▸), *Mercury* (Macrae *et al.*, 2008 ▸), *DIAMOND* (Brandenburg, 2006 ▸), *GOLD* (Chen, 2015 ▸), *MOPAC* (Stewart, 2013 ▸), *Crystal Explorer* (Wolff *et al.*, 2012 ▸), *publCIF* (Westrip, 2010 ▸) and *enCIFer* (Allen *et al.*, 2004 ▸).

## supplementary crystallographic information

### Crystal data

**Table:**

C_10_H_9_ClN_4_OS	*D*_x_ = 1.498 Mg m^−^^3^
*M**_r_* = 268.72	Mo *K*α radiation, λ = 0.71073 Å
Orthorhombic, *P*2_1_2_1_2_1_	Cell parameters from 9936 reflections
*a* = 6.2584 (1) Å	θ = 3.0–40.9°
*b* = 10.1734 (2) Å	µ = 0.48 mm^−^^1^
*c* = 18.7183 (3) Å	*T* = 200 K
*V* = 1191.78 (4) Å^3^	Prism, yellow
*Z* = 4	0.46 × 0.16 × 0.12 mm
*F*(000) = 552	

### Data collection

**Table:**

Bruker APEXII CCD area detector diffractometer	2295 reflections with *I* > 2σ(*I*)
Radiation source: fine-focus sealed X-ray tube, Bruker APEXII CCD	*R*_int_ = 0.013
φ and ω scans	θ_max_ = 26.0°, θ_min_ = 2.2°
Absorption correction: multi-scan (SADABS; Bruker, 2014)	*h* = −7→5
*T*_min_ = 0.697, *T*_max_ = 0.749	*k* = −12→12
10426 measured reflections	*l* = −23→23
2342 independent reflections	

### Refinement

**Table:**

Refinement on *F*^2^	Secondary atom site location: difference Fourier map
Least-squares matrix: full	Hydrogen site location: mixed
*R*[*F*^2^ > 2σ(*F*^2^)] = 0.020	H atoms treated by a mixture of independent and constrained refinement
*wR*(*F*^2^) = 0.056	*w* = 1/[σ^2^(*F*_o_^2^) + (0.0338*P*)^2^ + 0.2804*P*] where *P* = (*F*_o_^2^ + 2*F*_c_^2^)/3
*S* = 1.05	(Δ/σ)_max_ = 0.001
2342 reflections	Δρ_max_ = 0.19 e Å^−^^3^
167 parameters	Δρ_min_ = −0.15 e Å^−^^3^
0 restraints	Absolute structure: Flack *x* determined using 940 quotients [(*I*^+^)-(*I*^-^)]/[(*I*^+^)+(*I*^-^)] (Parsons *et al.*, 2013)
Primary atom site location: structure-invariant direct methods	Absolute structure parameter: 0.006 (9)

### Special details

**Table:**

Geometry. All esds (except the esd in the dihedral angle between two l.s. planes) are estimated using the full covariance matrix. The cell esds are taken into account individually in the estimation of esds in distances, angles and torsion angles; correlations between esds in cell parameters are only used when they are defined by crystal symmetry. An approximate (isotropic) treatment of cell esds is used for estimating esds involving l.s. planes.

### Fractional atomic coordinates and isotropic or equivalent isotropic displacement parameters (Å^2^)

**Table:**

	*x*	*y*	*z*	*U*_iso_*/*U*_eq_	
C1	0.4431 (3)	0.63210 (18)	0.56919 (9)	0.0229 (4)	
C2	0.3885 (3)	0.51360 (17)	0.61332 (9)	0.0200 (4)	
C3	0.5539 (3)	0.41669 (18)	0.59914 (9)	0.0199 (4)	
C4	0.5853 (3)	0.28884 (18)	0.62247 (9)	0.0215 (3)	
H4	0.487362	0.247309	0.653982	0.026*	
C5	0.7655 (3)	0.22425 (18)	0.59789 (9)	0.0246 (4)	
C6	0.9124 (3)	0.2840 (2)	0.55278 (10)	0.0278 (4)	
H6	1.036794	0.237337	0.538476	0.033*	
C7	0.8793 (3)	0.4117 (2)	0.52828 (10)	0.0271 (4)	
H7	0.977419	0.452902	0.496717	0.033*	
C8	0.6980 (3)	0.47585 (18)	0.55173 (9)	0.0226 (4)	
C9	−0.0829 (3)	0.58961 (18)	0.70690 (9)	0.0216 (4)	
C10	−0.2880 (3)	0.4484 (2)	0.78682 (11)	0.0345 (4)	
H10A	−0.260071	0.490692	0.832924	0.052*	
H10B	−0.301350	0.353251	0.793692	0.052*	
H10C	−0.421127	0.483020	0.766648	0.052*	
Cl1	0.80561 (8)	0.06052 (5)	0.62274 (3)	0.03753 (15)	
N1	0.6267 (3)	0.60279 (16)	0.53459 (8)	0.0254 (3)	
H1N	0.684 (4)	0.652 (2)	0.5082 (14)	0.037 (7)*	
N2	0.2289 (3)	0.49844 (14)	0.65623 (7)	0.0210 (3)	
N3	0.0946 (3)	0.60072 (16)	0.66389 (8)	0.0236 (3)	
H3N	0.110 (4)	0.669 (2)	0.6405 (12)	0.031 (6)*	
N4	−0.1123 (3)	0.47545 (16)	0.73815 (8)	0.0244 (3)	
H4N	−0.014 (4)	0.415 (3)	0.7332 (13)	0.040 (7)*	
O1	0.3382 (2)	0.73458 (13)	0.56517 (7)	0.0295 (3)	
S1	−0.24203 (8)	0.72186 (5)	0.71392 (3)	0.03074 (13)	

### Atomic displacement parameters (Å^2^)

**Table:**

	*U*^11^	*U*^22^	*U*^33^	*U*^12^	*U*^13^	*U*^23^
C1	0.0266 (9)	0.0228 (9)	0.0194 (8)	−0.0015 (7)	−0.0011 (7)	0.0016 (7)
C2	0.0213 (8)	0.0188 (8)	0.0198 (8)	0.0008 (7)	−0.0020 (6)	0.0005 (6)
C3	0.0189 (8)	0.0235 (9)	0.0174 (7)	−0.0005 (7)	−0.0008 (6)	−0.0016 (7)
C4	0.0212 (8)	0.0240 (9)	0.0194 (7)	0.0020 (7)	0.0004 (7)	0.0010 (7)
C5	0.0249 (9)	0.0249 (8)	0.0241 (8)	0.0059 (9)	−0.0053 (7)	−0.0014 (7)
C6	0.0201 (9)	0.0358 (10)	0.0275 (9)	0.0053 (8)	0.0005 (7)	−0.0074 (8)
C7	0.0221 (9)	0.0352 (10)	0.0240 (8)	−0.0047 (8)	0.0051 (7)	−0.0038 (8)
C8	0.0245 (10)	0.0248 (9)	0.0185 (7)	−0.0041 (7)	−0.0002 (7)	−0.0022 (6)
C9	0.0178 (8)	0.0240 (9)	0.0231 (8)	0.0004 (7)	−0.0022 (7)	−0.0042 (7)
C10	0.0261 (10)	0.0391 (11)	0.0382 (10)	−0.0029 (9)	0.0065 (9)	0.0058 (9)
Cl1	0.0386 (3)	0.0307 (3)	0.0433 (3)	0.0154 (2)	−0.0012 (2)	0.0053 (2)
N1	0.0305 (9)	0.0239 (8)	0.0219 (7)	−0.0032 (7)	0.0054 (6)	0.0032 (6)
N2	0.0202 (8)	0.0197 (6)	0.0230 (7)	0.0018 (6)	−0.0012 (6)	−0.0014 (5)
N3	0.0239 (8)	0.0193 (7)	0.0277 (8)	0.0042 (6)	0.0044 (6)	0.0017 (6)
N4	0.0192 (8)	0.0246 (8)	0.0294 (8)	0.0016 (7)	0.0017 (6)	0.0008 (6)
O1	0.0366 (8)	0.0230 (7)	0.0289 (6)	0.0061 (6)	−0.0009 (6)	0.0066 (5)
S1	0.0266 (2)	0.0249 (2)	0.0407 (3)	0.0070 (2)	0.0042 (2)	−0.00341 (19)

### Geometric parameters (Å, º)

**Table:**

C1—O1	1.234 (2)	C7—H7	0.9500
C1—N1	1.352 (3)	C8—N1	1.404 (2)
C1—C2	1.501 (2)	C9—N4	1.313 (2)
C2—N2	1.291 (2)	C9—N3	1.376 (2)
C2—C3	1.454 (2)	C9—S1	1.6792 (18)
C3—C4	1.386 (3)	C10—N4	1.455 (2)
C3—C8	1.401 (3)	C10—H10A	0.9800
C4—C5	1.384 (3)	C10—H10B	0.9800
C4—H4	0.9500	C10—H10C	0.9800
C5—C6	1.388 (3)	N1—H1N	0.79 (3)
C5—Cl1	1.7475 (19)	N2—N3	1.345 (2)
C6—C7	1.393 (3)	N3—H3N	0.83 (3)
C6—H6	0.9500	N4—H4N	0.87 (3)
C7—C8	1.381 (3)		
			
O1—C1—N1	127.53 (18)	C7—C8—N1	128.59 (18)
O1—C1—C2	126.21 (17)	C3—C8—N1	109.59 (16)
N1—C1—C2	106.26 (16)	N4—C9—N3	116.50 (16)
N2—C2—C3	125.68 (16)	N4—C9—S1	126.19 (14)
N2—C2—C1	127.93 (16)	N3—C9—S1	117.30 (13)
C3—C2—C1	106.39 (15)	N4—C10—H10A	109.5
C4—C3—C8	120.74 (17)	N4—C10—H10B	109.5
C4—C3—C2	132.84 (17)	H10A—C10—H10B	109.5
C8—C3—C2	106.41 (16)	N4—C10—H10C	109.5
C5—C4—C3	117.17 (17)	H10A—C10—H10C	109.5
C5—C4—H4	121.4	H10B—C10—H10C	109.5
C3—C4—H4	121.4	C1—N1—C8	111.32 (16)
C4—C5—C6	122.28 (18)	C1—N1—H1N	123.1 (19)
C4—C5—Cl1	118.75 (15)	C8—N1—H1N	125.5 (19)
C6—C5—Cl1	118.95 (15)	C2—N2—N3	117.21 (15)
C5—C6—C7	120.64 (18)	N2—N3—C9	120.21 (15)
C5—C6—H6	119.7	N2—N3—H3N	121.4 (17)
C7—C6—H6	119.7	C9—N3—H3N	118.2 (17)
C8—C7—C6	117.32 (17)	C9—N4—C10	123.53 (17)
C8—C7—H7	121.3	C9—N4—H4N	118.4 (17)
C6—C7—H7	121.3	C10—N4—H4N	117.8 (17)
C7—C8—C3	121.81 (18)		
			
O1—C1—C2—N2	−2.4 (3)	C6—C7—C8—N1	−179.15 (17)
N1—C1—C2—N2	178.37 (18)	C4—C3—C8—C7	−2.1 (3)
O1—C1—C2—C3	178.15 (18)	C2—C3—C8—C7	178.33 (16)
N1—C1—C2—C3	−1.04 (19)	C4—C3—C8—N1	177.97 (16)
N2—C2—C3—C4	2.7 (3)	C2—C3—C8—N1	−1.55 (19)
C1—C2—C3—C4	−177.88 (19)	O1—C1—N1—C8	−179.07 (18)
N2—C2—C3—C8	−177.86 (17)	C2—C1—N1—C8	0.1 (2)
C1—C2—C3—C8	1.57 (19)	C7—C8—N1—C1	−178.94 (18)
C8—C3—C4—C5	1.1 (2)	C3—C8—N1—C1	0.9 (2)
C2—C3—C4—C5	−179.57 (18)	C3—C2—N2—N3	178.33 (16)
C3—C4—C5—C6	1.1 (3)	C1—C2—N2—N3	−1.0 (3)
C3—C4—C5—Cl1	−177.22 (13)	C2—N2—N3—C9	177.74 (16)
C4—C5—C6—C7	−2.3 (3)	N4—C9—N3—N2	−0.8 (2)
Cl1—C5—C6—C7	176.06 (14)	S1—C9—N3—N2	179.76 (13)
C5—C6—C7—C8	1.2 (3)	N3—C9—N4—C10	178.17 (17)
C6—C7—C8—C3	1.0 (3)	S1—C9—N4—C10	−2.4 (3)

### Hydrogen-bond geometry (Å, º)

Cg is the centroid of the C3–C8 ring.

**Table:**

*D*—H···*A*	*D*—H	H···*A*	*D*···*A*	*D*—H···*A*
N3—H3*N*···O1	0.83 (2)	2.12 (3)	2.756 (2)	134 (2)
N1—H1*N*···O1^i^	0.79 (2)	2.04 (3)	2.824 (2)	175 (2)
N4—H4*N*···S1^ii^	0.88 (3)	2.72 (3)	3.518 (2)	152 (2)
C6—H6···*Cg*^iii^	0.95	2.61	3.410 (2)	142

Symmetry codes: (i) *x*+1/2, −*y*+3/2, −*z*+1; (ii) −*x*, *y*−1/2, −*z*+3/2; (iii) *x*+1/2, −*y*+1/2, −*z*+1.
